# Diagnostic accuracy of a velcro sound detector (VECTOR) for interstitial lung disease in rheumatoid arthritis patients: the InSPIRAtE validation study (INterStitial pneumonia in rheumatoid ArThritis with an electronic device)

**DOI:** 10.1186/s12890-019-0875-x

**Published:** 2019-06-20

**Authors:** A. Manfredi, G. Cassone, S. Cerri, V. Venerito, A. L. Fedele, M. Trevisani, F. Furini, O. Addimanda, F. Pancaldi, G. Della Casa, R. D’Amico, R. Vicini, G. Sandri, P. Torricelli, I. Celentano, A. Bortoluzzi, N. Malavolta, R. Meliconi, F. Iannone, E. Gremese, F. Luppi, C. Salvarani, M. Sebastiani, on behalf of GISEA (Gruppo Italiano Studio Early Arthritis)

**Affiliations:** 1 Rheumatology Unit, Azienda Policlinico di Modena, University of Modena and Reggio Emilia, Via del Pozzo, 71 41121 Modena, Italy; 2 Respiratory Disease Unit, Azienda Policlinico di Modena, University of Modena and Reggio Emilia, 41124 Modena, Italy; 3 Rheumatology Unit, Dipartimento Interdisciplinare di Medicina, University of Bari, 70124 Bari, Italy; 4 Rheumatology Unit, Catholic University of the Sacred Heart, 00168 Rome, Italy; 5 Rheumatology Unit, Azienda Ospedaliero-Universitaria di Bologna Policlinico Sant’Orsola-Malpighi, 40121 Bologna, Italy; 6 Rheumatology Unit, Clinical and experimental Medicine, Sant’Anna Hospital, 44121 Ferrara, Italy; 7 Rheumatology Unit, Department of Biomedical and Neuromotor Sciences, Rizzoli Orthopaedic Institute and University of Bologna, 40136 Bologna, Italy; 8 Department of Sciences and Methods for Engineering, University of Modena and Reggio Emilia, 41124 Modena, Italy; 9 Radiology Unit, Azienda Policlinico di Modena, University of Modena and Reggio Emilia, 41124 Modena, Italy; 10 Unit of Statistics in Medicine, Department of Oncology and Hematology, University of Modena and Reggio Emilia, 41124 Modena, Italy; 11 Rheumatology Unit, Santa Maria Hospital, IRCCS, 42121 Reggio Emilia, Italy

**Keywords:** Rheumatoid arthritis, Interstitial lung disease, Velcro sound, Diagnostic accuracy

## Abstract

**Background:**

Interstitial lung disease (ILD) is a severe systemic manifestation of rheumatoid arthritis (RA). High-resolution computed tomography (HRCT) represents the gold standard for the diagnosis of ILD, but its routine use for screening programs is not advisable because of both high cost and X-ray exposure. Velcro crackles at lung auscultation occur very early in the course of interstitial pneumonia, and their detection is an indication for HRCT. Recently, we developed an algorithm (VECTOR) to detect the presence of Velcro crackles in pulmonary sounds and showed good results in a small sample of RA patients.

The aim of the present investigation was to validate the diagnostic accuracy of VECTOR in a larger population of RA patients, compared with that of the reference standard of HRCT, from a multicentre study.

**Methods:**

To avoid X-ray exposure, we enrolled 137 consecutive RA patients who had recently undergone HRCT. Lung sounds of all patients were recorded in 4 pulmonary fields bilaterally with a commercial electronic stethoscope (ES); subsequently, all HRCT images were blindly evaluated by a radiologist, and audio data were analysed by means of VECTOR.

**Results:**

Fifty-nine of 137 patients showed ILD (43.1%). VECTOR correctly classified 115/137 patients, showing a diagnostic accuracy of 83.9% and a sensitivity and specificity of 93.2 and 76.9%, respectively.

**Conclusions:**

VECTOR may represent the first validated tool for the screening of RA patients who are suspected for ILD and who should be directed to HRCT for the diagnosis.

Moreover, early identification of RA-ILD could contribute to the design of prospective studies aimed at elucidating unclear aspects of the disease.

## Background

Rheumatoid arthritis (RA) is a chronic inflammatory disease secondary to immune system dysfunction, characterized by synovial joint swelling and tenderness with joint destruction and progressive disability. RA is often complicated by extra-articular manifestations, and interstitial lung disease (ILD) is one of the most frequent and deleterious complications with a negative impact on both the therapeutic approach and overall prognosis [1–4].

Approximately 10% of the RA population develops clinically significant ILD that is responsible for not only decreased quality of life and progressive chronic disability but also for 10–20% of deaths associated with the disease, with a mean survival of 5–8 years [5, 6].

Since no controlled studies are available, the therapeutic approach to RA-ILD is still debated and further complicated by the supposed role of many conventional and biologic disease-modifying anti-rheumatic drugs (DMARDs) in the onset or worsening of pre-existing ILD [7, 8]. In this regard, the British Society of Rheumatology has specifically cautioned prescribing TNFi to patients with RA-ILD, while in 2008, the American College of Rheumatology (ACR) contraindicated methotrexate for the treatment of these patients [9]. In fact, current literature data are not able to fill the gap of knowledge in this intriguing matter. Considering the possible severity of this complication and the therapeutic implications, investigating a better approach to obtain an early diagnosis is mandatory.

High-resolution computed tomography (HRCT) represents the gold standard for the diagnosis of this extra-articular manifestation, but ILD can appear in any stage of RA, entailing the need for a systematic assessment of lung involvement, and routine use of HRCT for screening programs is not advisable for both high cost and X-ray exposure [10].

In this background, a delayed diagnosis could be responsible for possible severe complications; therefore, the use of reliable and non-invasive tools may improve early diagnosis and better management of the disease.

A Velcro sound is a pulmonary sound defined as a fine crackle that is soft and short in duration, similar to the sound heard when the joined strips of jogging shoes are slowly separated. The detection of this typical sound, generally present throughout the inspiratory time and persisting after several deep breaths, has been proposed as an easy and repeatable screening for the early diagnosis of idiopathic pulmonary fibrosis and other forms of ILD [11, 12]. Recently, we developed an algorithm, named VECTOR (VElcro Crackles detecTOR), to detect the presence of Velcro crackles in pulmonary sounds recorded by an electronic stethoscope (ES) and that showed good results in a low sample of RA patients. In this preliminary study, the diagnostic accuracy of VECTOR was 90%, with a sensitivity of 92.6% and a specificity of 88.4% [13].

The aim of the present study was to validate the diagnostic accuracy of VECTOR in a larger population of RA patients, compared with that of the reference standard of HRCT, from a multicentre study.

## Patients and methods

The InsPIRAtE (INterStitial Pneumonia in Rheumatoid ArThritis with an Electronic device) study involved seven Italian tertiary rheumatologic centres with clinical experience in rheumatic disorders and interstitial lung diseases after approval from the local ethical committee. All patients were evaluated by means of VECTOR, and the results (presence or absence of Velcro crackles) were compared in a blind manner with HRCT (presence or absence of ILD).

### Inclusion criteria

All consecutive RA patients, classified according to 1987 or 2010 ACR classification criteria [14, 15], with a recent HRCT evaluation were eligible for the study and enrolled in a six-month period. According to clinical history, HRCT should have been performed within 12 months in the absence of the subsequent appearance or variation of signs or symptoms suggestive of lung disease (cough, dyspnoea, Velcro sound at routine clinical examination).

The reason for HRCT prescription was not a selection criterion for participation in the study.

### Exclusion criteria

Exclusion criteria were represented by:

- significant variations in respiratory symptoms after HRCT imaging (when possible, a new HRCT was requested);
- presence of pleural effusion or pneumothorax at HRCT;
- an overlap diagnosis with connective tissue disease.

### Study design

**Fig. 1:**
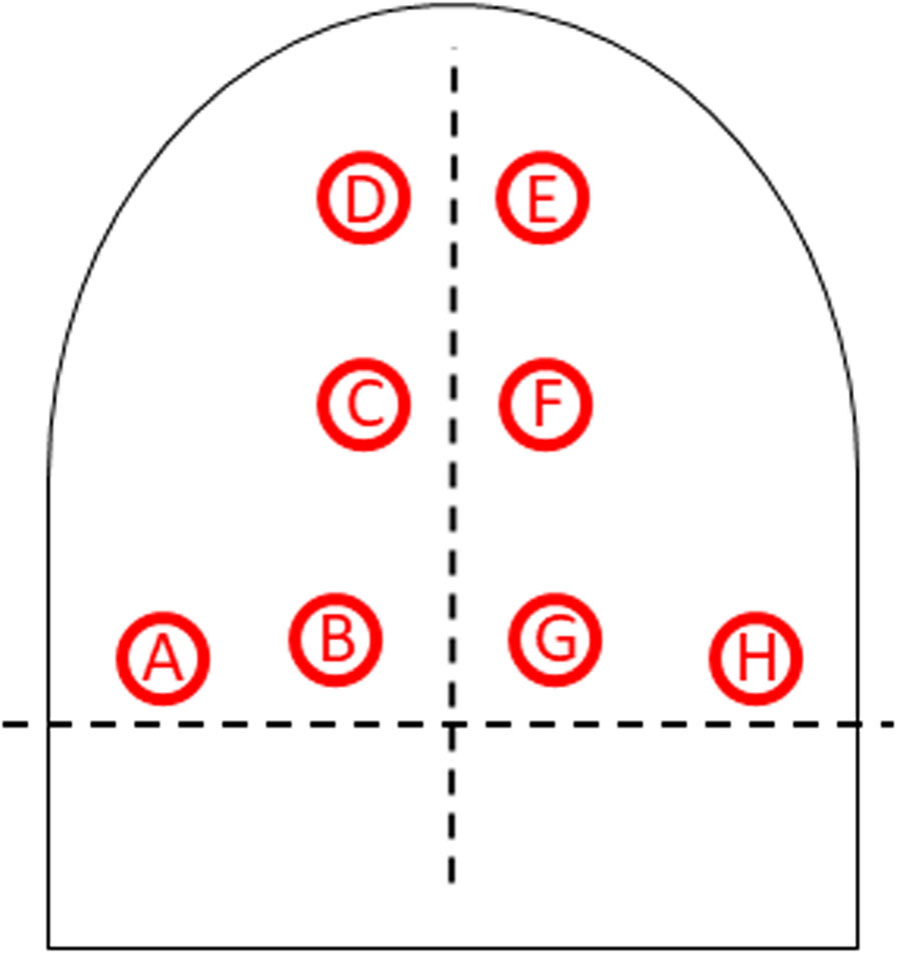
All patients were auscultated bilaterally in 4 pulmonary fields at the dorsal level: 2 at the basal field, 1 at the middle field and 1 at the upper field

According to our previous experience, respiratory sounds were recorded in 4 pulmonary fields bilaterally (2 at the basal field, 1 at the middle field and 1 at the upper field; see Fig. 1) in a silent environment with a commercial ES (Littmann 3200™ 3 M, USA). Then, audio files acquired for each patient were digitized, coded, saved as a WAV file and analysed by mean of VECTOR.

Moreover, all HRCT images were transferred on DICOM format, anonymized, coded and evaluated in a blind manner by an expert thoracic radiologist for the assessment of ILD.

Moreover, for all patients, the value of forced vital capacity (FVC), diffusion capacity of the lungs for CO (DLCO), the results of thoracic X-rays, the presence of Velcro crackles during thoracic examination and the presence of cough or dyspnoea at baseline were recorded.

#### Assessment of lung involvement

##### Radiological

All HRCT exams were performed using different multidetector scanners with a slice thickness of less than 2 mm, from the lung apices to below the costophrenic angles, reconstructed using an edge-enhancing algorithm. The scan was performed in the supine position at full inspiration. All images were viewed at a window setting optimized for assessment of the lung parenchyma (width 1500 HU; level − 700 HU). HRCT scans were assessed by an expert chest radiologist who interpreted the radiologic pattern of ILD according to American Thoracic Society, European Respiratory Society, Japanese Respiratory Society and Latin American Thoracic Association statement on the diagnosis of idiopathic pulmonary fibrosis (IPF) [16, 17]. The pattern of disease was recorded as definite, possible and inconsistent with usual interstitial pneumonia (UIP) (see Table 1). If an inconsistent UIP pattern was noted, we specified whether it was compatible with nonspecific interstitial pneumonia (NSIP), organizing pneumonia (OP) or lymphoid interstitial pneumonia (LIP) [18, 19]. When radiologic findings were not suggestive of any ILD, the radiologist recorded the presence of nodules, pleural effusion or other isolated manifestations of pulmonary disease such as consolidation.

**Table 1:** Clinical and serological features of 137 patients with rheumatoid arthritis

	Total	ILD -	ILD +	p
Nr.	137	78	59	
Smoke (%)	37.2	39.7	34	ns
Sex M/F	1/1.83	1/2.54	1/1.31	ns
ACPA (%)	77.2	77.1	77.2	ns
Rheumatoid factor (%)	78.6	81.1	75.4	ns
Forced vital capacity (% ± SD)	91.8 ± 22.3	93.1 ± 21.3	90.3 ± 23.7	ns
DLCO (% ± SD)	59.9 ± 18.0	65.7 ± 20.4	54.4 ± 13.6	0.015
Disease duration (years±SD)	11.1 ± 9.5	10.4 ± 7.8	12.1 ± 11.3	ns
Mean age at disease onset (years±SD)	56.1 ± 12.9	55.3 ± 12.3	57.0 ± 13.6	ns
Mean age at study entry (years±SD)	67.9 ± 9.9	66.5 ± 10.3	69.8 ± 9.1	0.049

*ILD* Interstitial lung disease, *M* Males, *F* Females, *ACPA* Anti-citrullinated peptide antibodies, *DLCO* Diffusion lung capacity of CO, *SD* Standard deviation

##### Functional

The results of pulmonary function tests were expressed as the percentage of the predicted value of each parameter and corrected for age, gender and height. Pulmonary function was considered abnormal if FVC was < 75% of predicted values. Single breath DLCO was used to assess gas transfer. A cut-off of 47% was chosen to identify a severe reduction in DLCO according to previous data [20].

##### Statistical analysis

Data were analysed using STATA statistical software (version 11, StataCorp LP, College Station, Texas, USA). Categorical variables were analysed by a chi square test, and differences between the means were determined using the Mann-Whitney or Student t-test for unpaired samples. Accordingly, the diagnostic accuracy, specificity and sensitivity of the clinical and instrumental diagnostic tools were calculated. *P* values ≤0.05 were considered statistically significant [21].

## Results

### General data

One hundred forty RA patients were enrolled in the study. Three patients were excluded for a low quality of recorded lung sounds. The indications for the HRCT were dyspnoea accompanied or not by fine crackles at lung auscultation (25.4%), a suggestive thorax X-ray (17.4%), the presence of fine crackles at lung auscultation (12.3%) and cough (4.3%). The remaining 56 patients underwent HRCT for other reasons (i.e., monitoring of lung nodules, infections, and screening for tumour or other lung diseases).

The clinical and serological features of the 137 patients investigated are reported in Table 1. ILD was detected in 59 patients (43.1%). No differences were observed between patients with or without ILD with regard to sex, autoantibodies, smoking habit, spirometry (FVC), mean age at disease onset, and disease duration. In contrast, patients with ILD were older and showed a lower DLCO than non-ILD patients (Table 1).

### Diagnostic accuracy of VECTOR

VECTOR correctly classified 115/137 patients (83.9%), showing a sensitivity and specificity of 93.2 and 76.9%, respectively. Only 4/59 patients with ILD were not identified by VECTOR, while false positive cases were 18/78. The diagnostic accuracy of VECTOR for detecting ILD was higher than that for detecting dyspnoea (64.6%), cough (58.3%), DLCO (54.9%), FVC (52.8%) and chest X-ray (71.3%) (see Table 2). The rheumatologist (GC, not blinded) correctly identified crackles in 69.1% of patients with ILD (versus 93.2% by using VECTOR) but also detected crackles in 34.2% of patients without ILD (versus 23.1% by using VECTOR).

**Table 2:** Diagnostic accuracy of clinical and instrumental variables

	Total	Diagnostic accuracy	Specificity	Sensitivity
Dyspnoea	29.1	64.6	81.3	41.2
Dry cough	12.1	58.3	89.2	15.1
Thoracic X-ray	35.1	71.3	80	57.8
DLCO < 47%	26	54.9	80	30.8
FVC < 70%	18.9	52.8	82.1	20
Velcro crackles^a^	49.2	67.2	65.7	69.1
VECTOR	52.9	83.9	76.9	93.2

^a^presence of Velcro crackles according to rheumatologist’s auscultation (AM)

VECTOR correctly identified patients with ILD despite their radiological pattern (Table 3). The 4 false negative patients showed an OP in two cases and an UIP pattern in the other two. The 2 patients with UIP patterns had advanced and clinically evident lung fibrosis, with difficulty properly breathing deeply. Patients with OP had a non-typical distribution of lung involvement that could interfere with proper auscultation. The possible patchy distribution of OP could explain the missing detection of these patients. Regarding false positive cases, 12/18 showed an airway disease, mainly bronchiectasis chronic bronchitis and obliterative bronchiolitis.

**Table 3:** Diagnostic accuracy according to the HRCT pattern

	Nr	%	Diagnostic accuracy (%)
UIP	30	21.7	93.5
NSIP	11	8	100
OP	8	5.8	75
LIP	1	0.7	100
Other	10	7.2	100
Normal	78	56.5	81.8

*HRCT* High resolution computerized tomography, *UIP* Usual interstitial pneumonia, *OP* Organizing pneumonia, *LIP* Lymphocytic interstitial pneumonia, *NSIP* Nonspecific interstitial pneumonia

The diagnostic accuracy of VECTOR was not influenced by the duration of lung disease or by the extension of lung involvement.

## Discussion

The present multicentre study confirmed the high sensitivity, specificity and diagnostic accuracy of VECTOR for detecting the presence of ILD in patients with RA.

RA-ILD is a field of great interest for both rheumatologists and pulmonologists. In recent years, many studies have been conducted to elucidate different facets of this harmful clinical problem, but they are all retrospective, with substantial bias due to diagnostic methodologies [22–25].

An early diagnosis of RA-ILD is mandatory since it represents one of the most severe and challenging extra-articular manifestations in RA patients, associated with very low quality of life and poor overall prognosis [1–4]. Given its significant impact, there is a need to develop strategies to increase the diagnosis of ILD before symptom occurrence. Moreover, ILD can occur at any stage of the disease; for this reason, a lung evaluation should always be included in the clinical assessment of RA patients, regardless of the disease duration or activity.

Currently, screening for RA-ILD is not feasible mainly because of the low diagnostic accuracy of any method other than HRCT, resulting in misdiagnosis or delayed diagnosis [6, 7, 10].

This limitation was already suggested in 2001 by Dawson et al., who showed the low diagnostic accuracy of clinical and functional features in RA-ILD patients. Among cases with HRCT evidence of ILD, only the presence of bilateral basal chest crackles was significantly associated with ILD, while restrictive pulmonary function tests and bilateral chest radiographic signs of ILD showed a very low diagnostic accuracy for ILD. Additionally, DLCO was associated with ILD, but 52% of patients had a reduced DLCO without ILD [26]. On the other hand, to study the possible risk factors for ILD appearance or progression, the natural evolution of lung disease and its prognosis, it is crucial to assess the whole clinical history of ILD and identify patients with subclinical lung involvement.

VECTOR could represent the first validated tool for the screening of RA patients suspected for ILD and who should be directed to HRCT for the diagnosis.

Our software, in combination with an ES, showed a sensitivity and specificity of 93.2 and 76.9%, respectively, and a very good diagnostic accuracy (83.9%), which is higher than any other method available to date, except for HRCT, but without any radiological exposure. The diagnostic accuracy of VECTOR was also higher than the capability of an expert rheumatologist to detect Velcro sounds by means of a pneumatic stethoscope, which reduced the prescription of needless HRCT and increased the diagnosis of ILD before clinical manifestations became evident. Of interest, in our population, less than half of the patients correctly identified by VECTOR showed clinical symptoms or a reduction in functional lung tests, confirming the usefulness of VECTOR in the identification of subclinical forms of ILD (Table 2).

RA is the most frequent inflammatory rheumatic disease with a high risk of developing ILD, but to date, a systematic approach to this problem is not routinely engaged in clinical practice [1, 5, 7, 27]. The proposed algorithm could represent an opportunity for all rheumatologists to improve the screening of patients to direct HRCT. It can be combined with an ES and allows real-time detection of Velcro crackles during a routine clinical examination.

The main limitation of our study is its retrospective design, which could justify a number of false positive cases, most likely due to transient conditions. Another possible explanation is the appearance of ILD subsequently to the HRCT used for the study. For example, in a patient with an apparent false positive result, an HRCT repeated after 10 months highlighted the presence of an initial ILD that was not visible in the previous evaluation.

We are aware that our population is not completely comparable with the general RA population, where we can suppose a lower rate of ILD but also of comorbidities that could affect the sensitivity and specificity of the test. We believe that sensitivity is a greater priority than specificity in the diagnosis of RA-ILD and that a possible reduction in specificity in a real-world setting could be justified by the correct identification of patients with ILD. However, although VECTOR performed well in the studied population, further studies should confirm its efficacy as a screening tool in patients with early lung disease and in the real-world setting of RA.

## Conclusion

VECTOR, associated with an ES, could allow a real-time screening of patients with ILD at rheumatologists, and it could be helpful in the design of prospective studies in which RA-ILD patients are identified with the help of this non-invasive method and followed during all disease courses.

## Abbreviations

ACR: American College of Rheumatology
DLCO: Diffusion capacity of the lungs for CO
ES: Electronic stethoscope
FVC: Forced vital capacity
HRCT: High-resolution computed tomography
ILD: Interstitial lung disease
IPF: Idiopathic pulmonary fibrosis
LIP: Lymphoid interstitial pneumonia
NSIP: Nonspecific interstitial pneumonia
OP: Organizing pneumonia
RA: Rheumatoid arthritis
UIP: Usual interstitial pneumonia
